# Supplementary material for: Magnon gap excitations in van der Waals antiferromagnet MnPSe3
Source: Sci Rep. 2024 Jul 30;14:17502. doi: 10.1038/s41598-024-67356-4 (PMC11289482; doi:10.1038/s41598-024-67356-4)
Supplement: Supplementary file 1 — Supplementary Information. [file 41598_2024_67356_MOESM1_ESM.pdf]

Supplemental Material for "Magnon gap excitations in van der Waals antiferromagnet $MnPSe_3$"

Dipankar Jana,[1,2,*] D. Vaclavkova,[1] I. Mohelsky,[1] P. Kapuscinski,[1,3] C. W. Cho,[1] I. Breslavetz,[1] M. Białek,[4,5] J.-Ph. Ansermet,[5] B. A. Piot,[1] M. Orlita,[1,6] C. Faugeras,[1] and M. Potemski[1,4,†]

[1] *Laboratoire National des Champs Magnétiques Intenses, LNCMI-EMFL, CNRS UPR3228, Univ. Grenoble Alpes, Univ. Toulouse, Univ. Toulouse 3, INSA-T, Grenoble and Toulouse, France*

[2] *Institute for Functional Intelligent Materials, National University of Singapore, 117544, Singapore*

[3] *Institute of Experimental Physics, Faculty of Physics, University of Warsaw, ul. Pasteura 5, PL-02-093 Warsaw, Poland*

[4] *CENTERA Labs, Institute of High Pressure Physics, PAS, 01-142 Warsaw, Poland*

[5] *Institute of Physics, Ecole Polytechnique Fédérale de Lausanne (EPFL), 1015 Lausanne, Switzerland*

[6] *Institute of Physics, Charles University, Ke Karlovu 5, Prague, 121 16, Czech Republic*

I. THz MONOCHROMATIC TRANSMISSION MEASUREMENTS

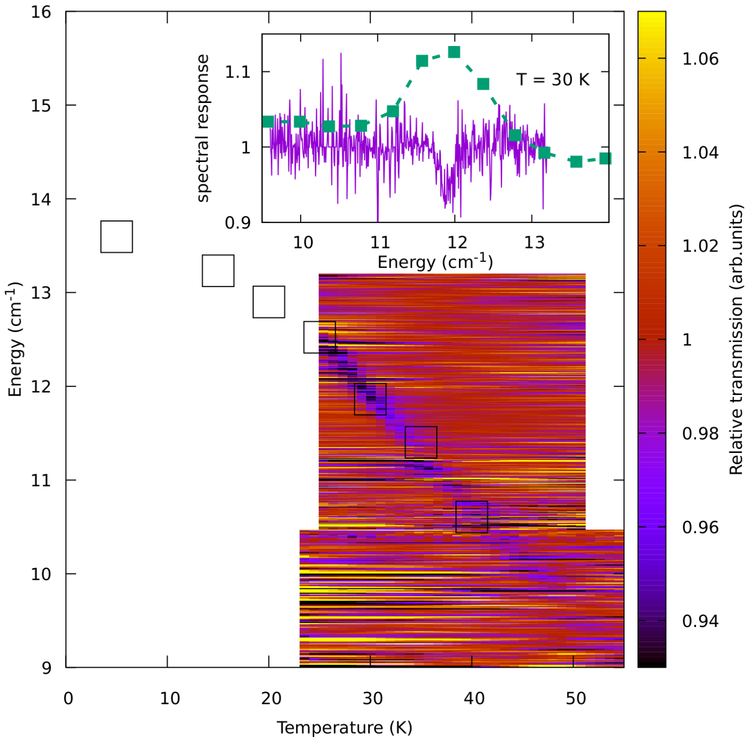

FIG. S1. False color map of normalised THz transmission spectra of $MnPSe_3$, at $B = 0\ T$, as a function of temperature in the spectral range from 9 to 13 cm^{-1}. A bulk sample of $MnPSe_3$ was placed on an aperture in a closed-cycle cryostat with optical windows. We used a monochromatic spectrometer based on frequency extenders to a vector network analyzer. See Ref. [S1] for the extended information on the experimental set-up applied. The energy positions of the upper magnon mode determined from Raman scattering measurements (see Fig. 1b. of the main text) are shown with open rectangles. An example of a (relative) transmission spectrum, measured at 30 K, is shown in the inset and compared to the corresponding Raman scattering signal shown with the dashed line. Linewidth determined using monochromatic THz spectroscopy data is about 0.1 cm^{-1}.

* jana.d02@nus.edu.sg
† marek.potemski@lncmi.cnrs.fr

II. RAMAN SCATTERING SPECTRA OF $MnPSe_3$, MEASURED AS A FUNCTION OF THE MAGNETIC FIELD APPLIED IN NEARLY OUT-OF-PLANE CONFIGURATION

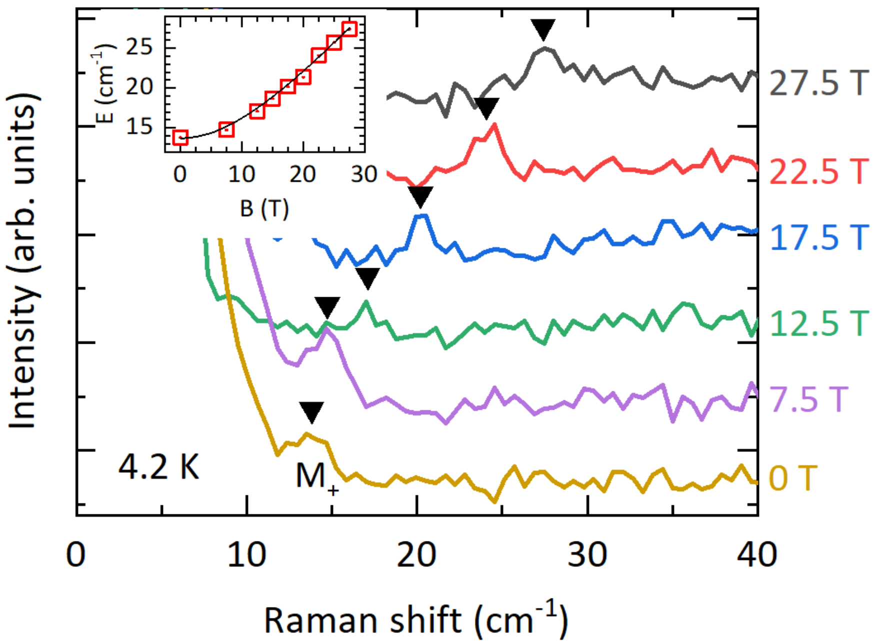

FIG. S2. Magneto-Raman scattering spectra of $MnPSe_3$, measured at 4.2 K, at several values of the magnetic field B. The B-field is applied at an angle of 20° with respect to the normal to the layers' plane. Only M_+ resonance could be traced in this experiment; the response due to the lower M_- magnon gap is hidden by the insufficiently rejected stray laser light. The dependence of the energy of the M_+ resonance upon the applied magnetic field is shown in the inset. As discussed in the main text, it follows the $w_{M+}(B) = \sqrt{(w_{M+})^2 + (g\mu_B B\cos(20°))^2}$ dependence as shwon by the solid line.

III. RAMAN SCATTERING SPECTRA OF $MnPSe_3$, MEASURED AS A FUNCTION OF THE IN-PLANE MAGNETIC FIELD (UP TO 30 T), IN A WIDE SPECTRAL RANGE.

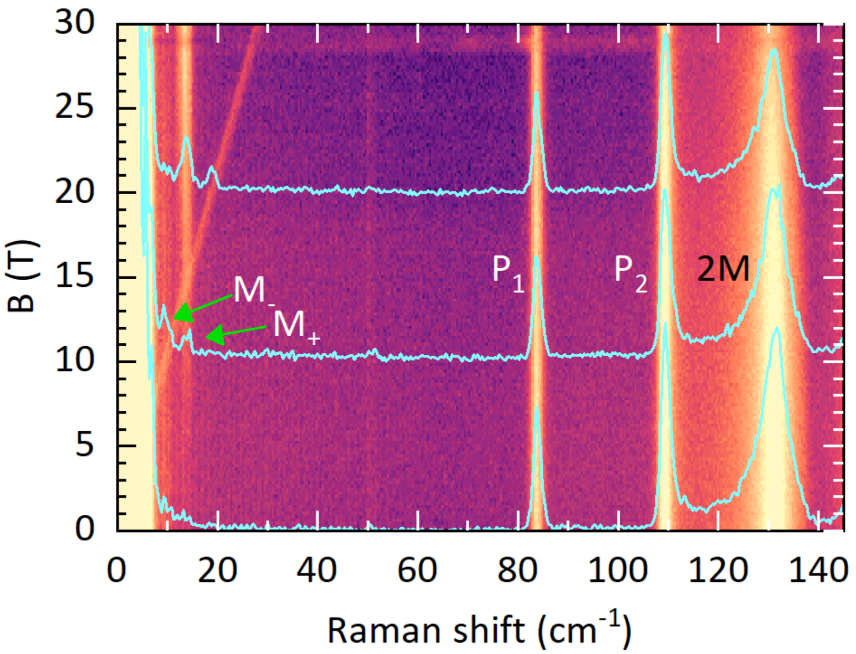

FIG. S3. False color map of Raman scattering spectra of $MnPSe_3$, measured at 4.2 K, as a function of the magnetic field applied in layers' a-b plane. A few representative spectra, measured at a magnetic field strength of 0 T, 10 T, and 20 T are also plotted. M_+ and M_- resonances correspond to the upper and lower magnon gap excitation which shows a crossover at around 14 T. P_1, P_2 correspond to phonon modes while 2M is due to two magnon excitation[S2].

IV. SIMULATION FOR THE IN-PLANE MAGNETIC FIELD DEPENDENCE OF MAGNON GAP EXCITATIONS IN A BIAXIAL $MnPSe_3$ ANTIFERROMAGNET

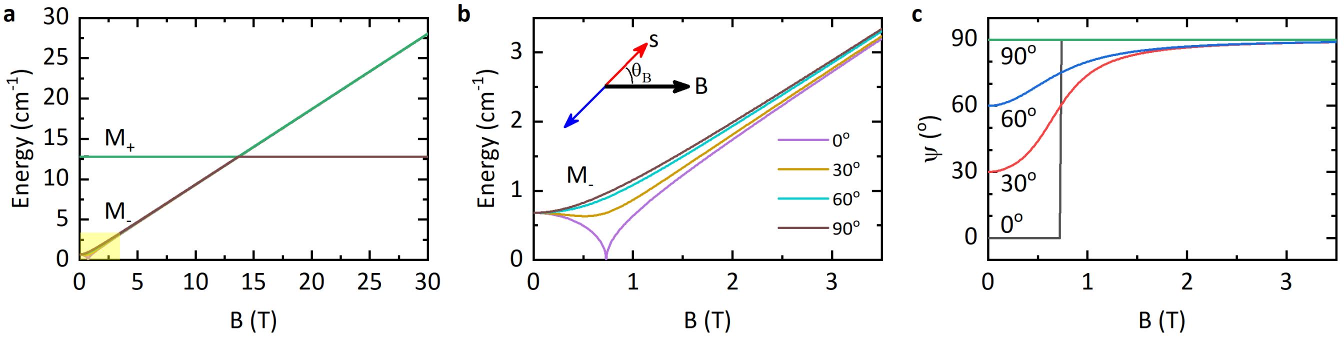

FIG. S4. (a) Simulated in-plane magnetic field dependence of low M_- and high M_+ energy magnon modes, in a broad range of magnetic fields, up to 30 T, for different θ_B angle between the direction of the applied magnetic field and the axis along which the spins are initially (at $B = 0$) aligned. (b) The dependence of M_- mode in the low field region as highlighted in Fig S5a for different θ_B, and (c) Upon the application of the in-plane magnetic field, the orientation of spins with respect to the direction of the magnetic field changes. As illustrated, the angle Ψ_B between the B direction and the axis of spin alignments evolves differently for different initial θ_B, angles.

V. SIMULATION FOR THE IN-PLANE MAGNETIC FIELD DEPENDENCE OF w_{M-} BY SUMMING UP THE CONTRIBUTION FROM DIFFERENT DOMAINS

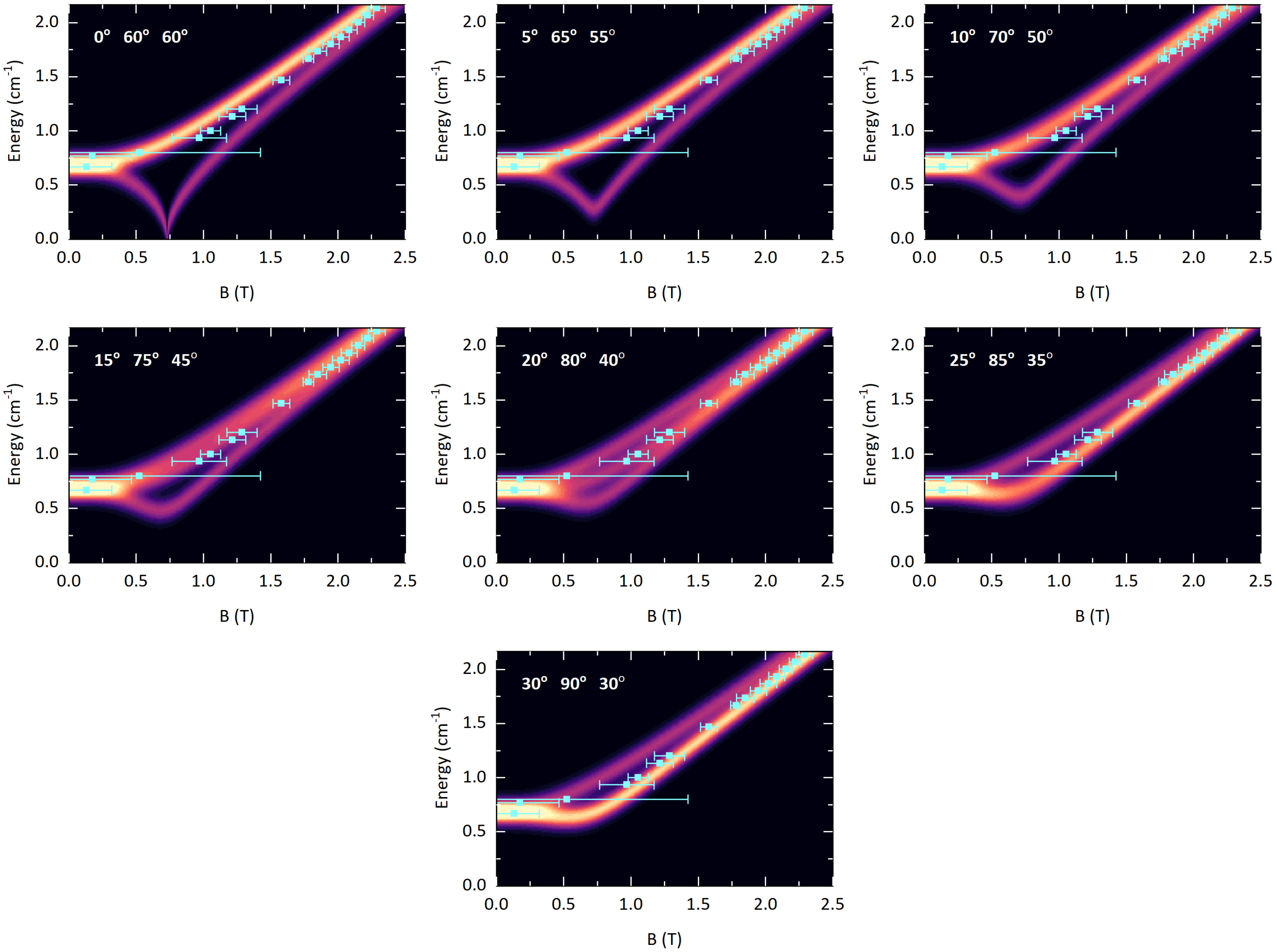

FIG. S5. False color maps of the simulated (normalized) magnetic field dependence of the intensity of M_- mode resonance absorption considering the three symmetry orientations of the spins in different domains. The hexagonal symmetry of the crystal and the antiferromagnetic ordering restricts the spin orientations of different domains to θ, (θ+60°), (θ-60°) with respect to the in-plane magnetic field. Different plots correspond to different directions of the in-plane magnetic field with respect to the one spin orientation (θ). Peak energies obtained from phase spectra (Fig. 3a of the main manuscript) are shown by cyan squares whereas the half-widths of the observed resonances are indicated with horizontal bars.

[S1] M. Białek, T. Ito, H. Rønnow, and J.-P. Ansermet, Terahertz-optical properties of a bismuth ferrite single crystal, Phys. Rev. B **99**, 064429 (2019).

[S2] T. T. Mai, K. F. Garrity, A. McCreary, J. Argo, J. R. Simpson, V. Doan-Nguyen, R. V. Aguilar, and A. R. H. Walker, Magnon-phonon hybridization in 2D antiferromagnet $MnPSe_3$, Sci. Adv. **7**, eabj3106 (2021).
